# Cavity-control of interlayer excitons in van der Waals heterostructures

**DOI:** 10.1038/s41467-019-11620-z

**Published:** 2019-08-16

**Authors:** Michael Förg, Léo Colombier, Robin K. Patel, Jessica Lindlau, Aditya D. Mohite, Hisato Yamaguchi, Mikhail M. Glazov, David Hunger, Alexander Högele

**Affiliations:** 1 Fakultät für Physik, Munich Quantum Center, and Center for NanoScience (CeNS), Ludwig-Maximilians-Universität München, Geschwister-Scholl-Platz 1, 80539 München, Germany; 2 Department of Chemical and Biomolecular Engineering, Rice University, Houston, TX 77005 USA; 3 Los Alamos National Laboratory (LANL), Los Alamos, NM 87545 USA; 4 Ioffe Institute, 26 Polytechnicheskaya, St. Petersburg, Russia 194021; 5 Spin Optics Laboratory, Saint Petersburg State University, 1 Ul’yanovskaya str., St. Petersburg, Russia 198504; 6 Physikalisches Institut, Karlsruher Institut für Technologie, Wolfgang-Gaede-Straße 1, 76131 Karlsruhe, Germany; 7 Munich Center for Quantum Science and Technology (MCQST), Schellingtraße 4, 80799 München, Germany

**Keywords:** Two-dimensional materials, Two-dimensional materials

## Abstract

Monolayer transition metal dichalcogenides integrated in optical microcavities host exciton-polaritons as a hallmark of the strong light-matter coupling regime. Analogous concepts for hybrid light-matter systems employing spatially indirect excitons with a permanent electric dipole moment in heterobilayer crystals promise realizations of exciton-polariton gases and condensates with inherent dipolar interactions. Here, we implement cavity-control of interlayer excitons in vertical MoSe_2_-WSe_2_ heterostructures. Our experiments demonstrate the Purcell effect for heterobilayer emission in cavity-modified photonic environments, and quantify the light-matter coupling strength of interlayer excitons. The results will facilitate further developments of dipolar exciton-polariton gases and condensates in hybrid cavity – van der Waals heterostructure systems.

## Abstract (web summary)

Cavity-enhanced light-matter interaction in the weak-coupling regime is known to result in Purcell enhancement. Here the authors demonstrate Purcell enhancement in the photoluminescence of vertical MoSe_2_-WSe_2_ heterostructures coupled to a micro-cavity and determine the light-matter coupling strength for interlayer excitons.

## Introduction

Semiconductor transition metal dichalcogenides (TMDs) exhibit remarkable optoelectronic and valleytronic properties in the limit of direct band-gap monolayer (MLs)^1–4^. High oscillator strength renders the materials ideal for the studies of collective strong-coupling phenomena mediated among excitons and photons by optical resonators^5^. This limit of new bosonic eigenstates of half-matter and half-light quasiparticles known as exciton-polaritons is routinely achieved for ML TMDs in various types of cavities^6–9^. In contrast, cavity-control of van der Waals heterobilayers (HBLs) has been elusive despite their potential for fundamental studies of dipolar gases with intriguing polarization dynamics upon expansion^10^ and condensation phenomena^11^. Composed of two dissimilar MLs in staggered band alignment^12,13^, such van der Waals heterostructures host layer-separated electron-hole pairs in response to optical excitation^14^. The spatial separation of Coulomb-correlated electrons and holes gives rise to a permanent exciton dipole moment along the stacking axis, and extended lifetimes up to hundreds of ns^14–17^. Although long lifetimes are beneficial for providing sufficient time scales for thermalization, finite exciton dipole moments ensure mutual interactions in exciton-polaritons gases and condensates. To date, however, the integration of HBLs into optical cavities has been impeded by the involved fabrication of exfoliation-stacked HBL systems which require careful alignment of both MLs along the crystallographic axes to reduce momentum mismatch between electrons and holes residing in dissimilar layers.

As opposed to exfoliation-stacking, chemical vapor deposition (CVD) realizes inherently aligned TMD heterostructures with atomically sharp interfaces both in lateral and vertical geometries^18,19^. However, even in the presence of inherent angular alignment, excitons in van der Waals stacks of incommensurate layers with dissimilar lattice constants are subject to moiré effects^20–24^ akin to twisted HBL systems^25^. As such, CVD-grown MoSe_2_-WS_2_ HBL with a lattice mismatch of a few percent feature moiré patterns with a period of ~10 nm^22^. In MoSe_2_-WSe_2_ heterostructures, on the other hand, the much smaller lattice mismatch of 0.1% can be accommodated by atomic vacancies to yield a fully commensurate HBL system free of moiré effects^26,27^ in nearly ideal R- and H-type stacking geometries^28^.

In our experiments we use such moiré-free vertical MoSe_2_-WSe_2_ HBL, synthesized by overgrowth of ML MoSe_2_ with ML WSe_2_, to demonstrate cavity-control of interlayer excitons. Our studies focus on the dynamics of HBL photoluminescence (PL) in weak coupling to a tunable optical micro-cavity. Akin to previous reports, the interlayer exciton PL from our sample exhibits rich spectral and temporal characteristics subject to competing interpretations with respect to the underlying origin and details^14–17^. We interpret our observations in the framework of interlayer excitons in various spin and valley configurations consistent with the theoretical framework of bright and dark excitons in commensurate HBLs. After establishing the signatures of interlayer excitons in continuous-wave and time-resolved PL spectroscopy and differential reflectance (DR), we present cavity-control of the respective HBL PL in a tunable micro-cavity configuration^29^. Specifically, we demonstrate Purcell enhancement in the light-matter interaction of interlayer excitons as evidenced by the simultaneous increase of their PL intensity and radiative decay rate, and quantify the respective light-matter coupling strength.

## Results

### Confocal photoluminescence spectroscopy of MoSe_2_-WSe_2_ heterobilayer

**Fig. 1:**
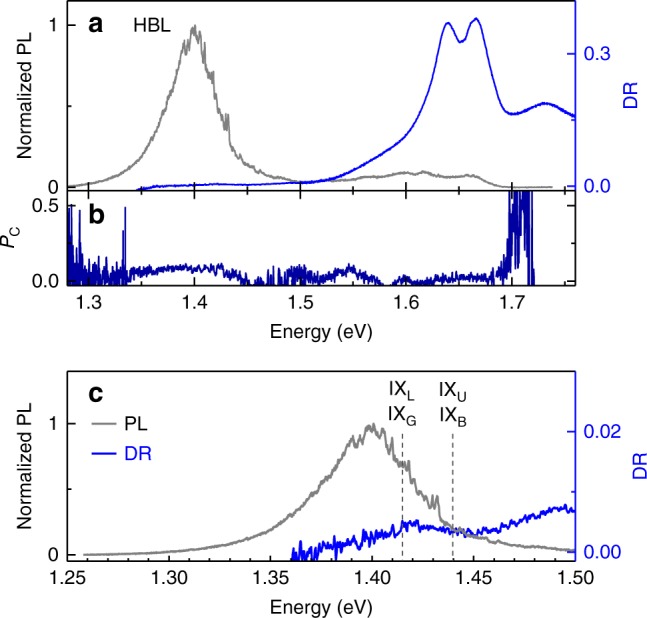
**a** Cryogenic PL (gray) and DR (blue) spectra of a vertical MoSe_2_-WSe_2_ stack with dominant features of the heterobilayer (HBL) peak in emission and intralayer excitons in absorption. **b** Degree of circular PL polarization, *P*_C_, under circularly polarized excitation. **c** Zoom-in to the intralayer exciton emission and absorption with energies of zero-momentum bright and gray excitons, IX_B_ and IX_G_, and momentum-dark spin-like and spin-unlike excitons, IX_L_ and IX_U_, indicated by dashed lines. The onset of absorption in the DR spectrum at ~1.37 eV stems from the inhomogenously broadened gray interlayer exciton state IX_G_

Before demonstrating cavity-control of HBL excitons, we discuss the main signatures of intralayer and interlayer optical transitions in cryogenic spectroscopy. Confocal PL and DR spectra of our MoSe_2_-WSe_2_ sample recorded at 4.2 K are shown in Fig. 1a. The DR spectrum at 1.65 and 1.75 eV is dominated by ML excitons in MoSe_2_ and WSe_2_, respectively. In PL, the MoSe_2_ ML contributes a pair of peaks ~1.65 eV stemming from neutral and charged intralayer excitons^30^. Consistent with previous studies of exfoliation-stacked heterostructures^14–17^, the cryogenic PL shows vanishingly small emission from intralayer WSe_2_ excitons and a strong low-energy peak of interlayer excitons around 1.40 eV. This HBL peak arises from photo-generated electrons and holes that relax over the conduction band (CB) and valence band (VB) offsets of ≤350 and 250 meV, respectively, to form interlayer excitons^14^.

The configuration of interlayer excitons in moiré-free HBL systems depends on the actual atomic registry. In the Supplementary Note 1, we provide a description of the exciton manifolds in both R- and H-type commensurate vertical HBLs for three types of distinct atomic registries as shown in the Supplementary Fig. 2. We note that the optical selection rules derived from symmetry considerations and summarized in the Supplementary Fig. 3 also hold locally for incommensurate heterostructures that feature different atomic registries over extended regions of moiré superlattices^31^. The HBL sample in our experiment corresponds to AB stacking in H-type registry with a rotation angle of multiples of 60° between the two TMD layers. The assignment follows from second-harmonic generation (SHG) mapping with lower intensity on HBL regions as compared to the SHG signal of ML regions^32^. Moreover, the positive degree of circular polarization P_C_, shown in Fig. 1b, is consistent with MoSe_2_-WSe_2_ HBL in AB stacking^27^.

For this specific stacking, we obtain from our symmetry analysis two optically active zero-momentum interlayer excitons. Bright excitons, IX_B_, involve an unoccupied spin-up (spin-down) VB state in WSe_2_ at *K* (*K*′) and an occupied spin-up (spin-down) CB state in MoSe_2_ at *K*′ (*K*). The gray exciton manifold with a smaller oscillator strength due to its antiparallel spin configuration^31^, IX_G_, involves an unoccupied spin-up (spin-down) VB state in WSe_2_ at *K* (*K*′) and an occupied spin-down (spin-up) CB state in MoSe_2_ at *K*′ (*K*). These bright and gray exciton states, split by the CB spin-orbit splitting of MoSe_2_^33^ and degenerate with their respective time-reversal counterparts, contribute through their respective radiative decay channels to the HBL peak in Fig. 1.

In addition to zero-momentum interlayer excitons with dipolar-allowed optical transitions, finite-momentum interlayer excitons result from spin-like (IX_L_) combinations of unoccupied spin-up (spin-down) VB states in WSe_2_ at *K* (*K*′) and occupied spin-up (spin-down) CB states in MoSe_2_ at *K* (*K*′), as well as spin-unlike (IX_U_) combinations of unoccupied spin-up (spin-down) VB states in WSe_2_ at *K* (*K*′) and occupied spin-down (spin-up) CB states in MoSe_2_ at *K* (*K*′). These two doubly degenerate states IX_U_ and IX_L_ with non-zero center-of-mass momentum are resonant with IX_B_ and IX_G_, respectively, yet void of direct radiative decay pathways due to momentum conservation constraints.

With this notion of interlayer excitons, we interpret the HBL peak in Fig. 1 as arising from dipolar-allowed recombination of IX_B_ and IX_G_ excitons as well as from phonon-assisted emission from momentum-dark excitons IX_L_. The IX_U_ reservoir is assumed to be empty due to relaxation of the photoexcited population into energetically lower-lying states. Bright and gray excitons contribute zero-phonon line (ZPLs) emission at their bare energy. Momentum-indirect excitons, on the other hand, contribute to the PL spectrum as phonon sidebands downshifted from their bare energy IX_L_ by the energy of acoustic or optical phonons (and their higher order combinations) that compensate for momentum mismatch in the light-matter coupling and thus promote radiative decay^34,35^. The corresponding spectral decomposition of the HBL peak, provided in the Supplementary Note 3, yields the energies of IX_B_ and IX_U_ as indicated by the dashed lines in Fig. 1c and an inhomogeneous broadening of 40–55 meV. Alternatively, the asymmetric HBL peak can be interpreted as being composed of IX_B_ and IX_G_ emission and red-shifted localized excitons trapped in disorder potentials.

**Fig. 2:**
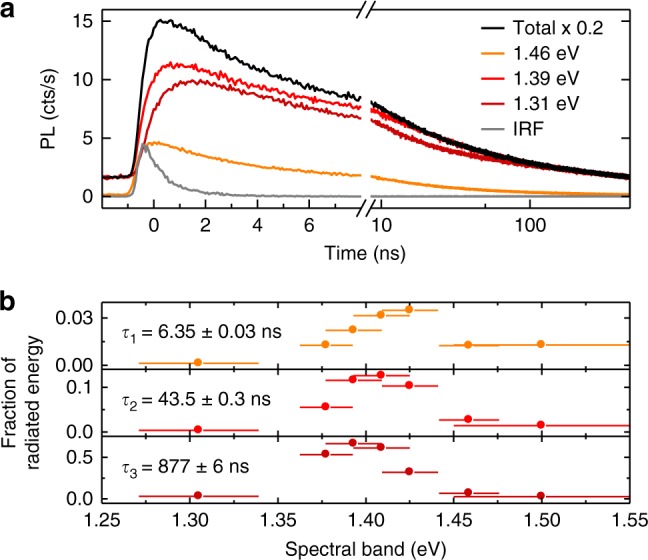
**a** Confocal PL decay measured in different spectral bands of the HBL peak in Fig. 1. Decay traces are shown in different colors for three spectral bands centered at 1.46, 1.39, and 1.31 eV together with the total decay trace in black (scaled by ×0.2) and the instrument response function (IRF) in gray. The decay of the total spectrally unfiltered PL was approximated best by three-exponential decay channels with time constants of ~6, 44, and 877 ns. **b** Relative contributions of the three decay channels in discrete spectral windows with central energies and widths represented by dots and bars, respectively. For each spectral window, the contributions were extracted from triple-exponential fits with decay constants fixed to the characteristic timescales of the total HBL emission

To substantiate the interpretation of the HBL peak as a convolution of IX_B_ and IX_G_ ZPLs and IX_L_ phonon sidebands, we carried out time-resolved PL experiments. Previous cryogenic studies of exfoliation-stacked MoSe_2_-WSe_2_ heterostructures reported interlayer excitons lifetimes in the range of 1–100 ns with single- or multi-exponential decay dynamics^10,14–17^. The spectrally broad interlayer HBL peak of our sample exhibited similar PL decay characteristics. The best approximation to the total HBL peak was obtained with three-exponential decay channels with lifetimes of ~6, 44, and 877 ns (see Supplementary Figs. 6 and 7 in the Supplementary Note 2). Consistent with our understanding of the HBL emission, the contributions of the individual decay channels to the total radiated PL energy varied significantly across the HBL peak. By performing PL decay measurements in narrow spectral windows at variable energies shown in Fig. 2a, we found that the relative weight of the slowest decay component with 877 ns decay constant increased at the expense of the more rapid components with 6 and 44 ns lifetimes as the spectral band of the measurement window was shifted to lower energies (Fig. 2b). In the red-most wing, interlayer PL was significantly delayed (note the prolonged rise-time of the PL traces in Fig. 2a recorded in the red wing) and dominated by the longest decay constant.

The cross-over from short to long PL lifetimes upon progressive red-shift provides support for our interpretation of the HBL peak. Our model predicts a decrease for the PL contribution from the momentum-bright exciton IX_B_ upon increasing red-shift from its ZPL, and this trend is consistently supported by the data in the upper panel of Fig. 2b. In this framework, the shortest decay channel is attributed to bright excitons IX_B_ (data in the upper panel of Fig. 2b), the intermediate timescale to gray excitons IX_G_ (central panel of Fig. 2b), and the long lifetime to phonon-assisted decay channels at larger red-shifts (lower panel of Fig. 2b). Alternatively, one could assign the fast and intermediate decay components to IX_B_ and IX_G_ decay channels, respectively, and the long decay component to defect-localized interlayer excitons.

### Purcell enhancement of MoSe_2_-WSe_2_ heterobilayer photoluminescence

**Fig. 3:**
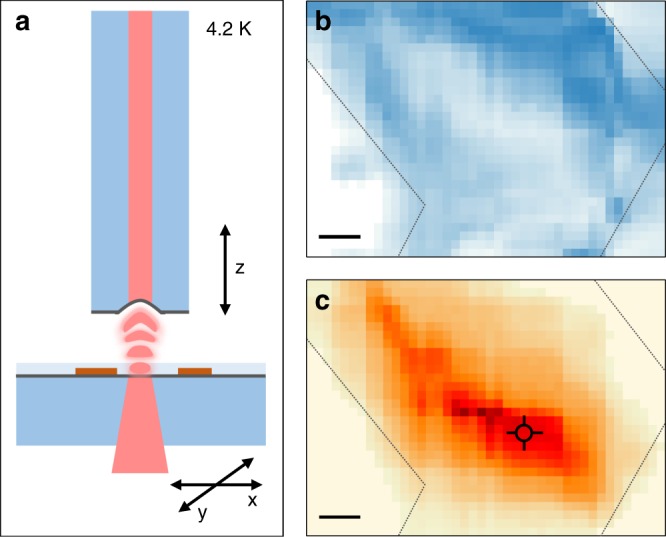
**a** Cavity setup at 4.2 K: the fiber-based micro-mirror forms the cavity together with a planar macro-mirror with CVD-grown MoSe_2_-WSe_2_ heterostructure on top. Independent translational degrees of freedom enable lateral sample displacement and cavity length detuning. **b** Transmission map recorded through the cavity with laser excitation at 635 nm (blue color corresponds to reduced transmission due to local variations in absorption and scattering). **c** Map of integrated PL intensity recorded simultaneously with the transmission map (dark red color represents maximum intensity). The cross indicates the position on the flake used in the measurements of Figs. 2 and 4, the gray dashed lines indicate the boundaries of the flake. The scale bar is 10 μm in both maps

In the following, we demonstrate cavity control of the HBL peak PL dynamics. To this end, we positioned a fiber micro-mirror above the macroscopic mirror with CVD-grown MoSe_2_-WSe_2_ flakes on top. The schematic drawing of the cavity setup with independent translational degrees of freedom along all three dimensions is shown in Fig. 3a. The related details of the cavity setup are described in the Supplementary Note 4 and include the transmission characteristics of the cavity as a function of variable cavity length in the Supplementary Fig. 9. Displacement of the sample mirror enabled coarse-tuning of the cavity length as well as two-dimensional positioning and profiling of the sample. The respective cryogenic transmission and PL maps of the HBL flake with PL data in Figs. 1 and 2 are shown in Fig. 3b, c.

The transmission map in Fig. 3b, recorded with the excitation laser at 635 nm, quantifies both absorption and scattering inside the cavity. The sizeable ML absorption in the range of several percent^36^ facilitated the detection of individual MLs and HBL via the cavity transmission. Scattering contrast at structural defects such as edges or transfer-related cracks provided additional guides to the identification of individual flakes. Equipped with the combined scanning capabilities and the data from transmission, it was straight forward to position the cavity into any point of interest on the HBL flake. In addition, by recording PL spectra at each raster scan-point of the cavity simultaneously with the transmission, PL intensity maps were obtained within the spectral band of interest, as shown for the interlayer exciton PL map of Fig. 3c.

**Fig. 4:**
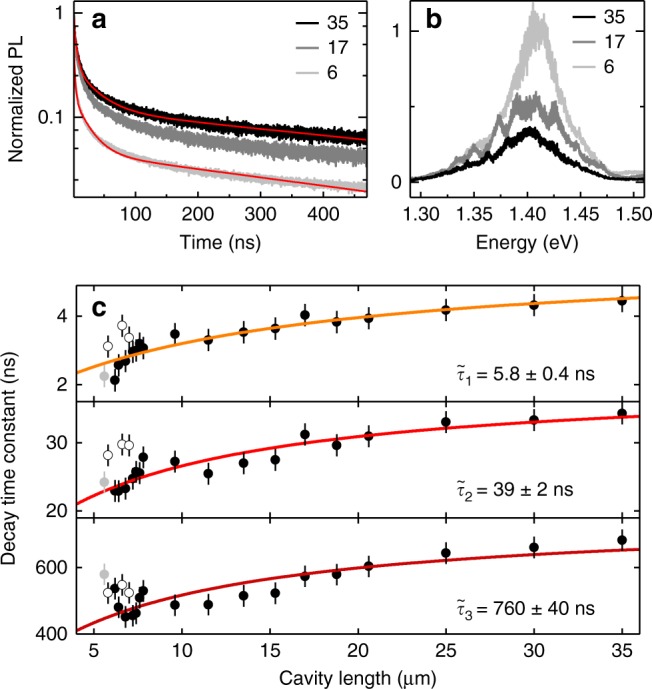
**a** Traces of interlayer exciton PL decay shown for three selected cavity lengths of 35, 17, and 6 μm. The solid lines are fits to the data with three-exponential decay constants. Note the speed up in the decay upon the reduction of the cavity length. **b** Spectra of interlayer exciton PL for the corresponding cavity lengths. **c** The evolution of the characteristic decay constants with the cavity length is shown by closed circles (error bars: least squares from best fit with three-exponential decay channels). The solid lines show model fits according to the theory of generalized Purcell enhancement. Open circles represent data where the cavity mode was spectrally detuned from the resonance with the interlayer peak; data shown in light gray were discarded from the fit procedure due to presumable physical contact between the fiber and the mirror

By monitoring both transmission and PL, we positioned the cavity on the spot indicated by the cross in Fig. 3c where the data of Figs. 1 and 2 were recorded with confocal spectroscopy, and performed PL decay measurements as a function of the cavity length. The respective decay traces are shown in Fig. 4a for cavity lengths of 35, 17, and 6 μm. Clearly, the PL decay speeds up with decreasing cavity length. This reduction of the characteristic lifetimes with decreasing cavity length was accompanied by an increase of the total PL intensity by a factor of 2.6 (Fig. 4b) as a hallmark of cavity-induced Purcell enhancement of excitonic emission. For a more quantitative analysis of Purcell enhancement, the PL traces recorded at different cavity lengths were modeled by a convolution of the instrument response function (IRF) and a three-exponential decay with amplitudes and time constants of each decay channel as free fit parameters, as described in the Supplementary Note 2. The corresponding model fits, shown as red solid lines in Fig. 4a, were used to extract the short, intermediate, and long decay time components for a given cavity length.

The respective set of data, shown in Fig. 4c, clearly demonstrates cavity-control of all three characteristic decay channels. The evolution of the lifetime shortening with decreasing cavity length is quantified by the ratio of the total decay rate in the cavity system *γ*_tot_ = *γ*_fs_ + *γ*_c_ to the bare free-space decay rate *γ*_fs_ as *γ*_tot_/*γ*_fs_ = 1 + *F*_p_, where *F*_P_ = *γ*_c_/*γ*_fs_ is the Purcell enhancement factor due to the cavity-modified decay rate *γ*_c_^37^. An estimate for the cavity-mediated Purcell enhancement can be obtained by identifying the values obtained from confocal PL dynamics with free-space lifetimes. Taking the smallest lifetime values for each decay channel from the data of Fig. 4c, this yields maximum measured Purcell factors *F*_P_ of 1.8 ± 0.3, 0.8 ± 0.1 and 0.9 ± 0.1 for the short, intermediate, and long lifetime components, respectively.

The difference in the Purcell factors is consistent with the different nature of the coupling between the corresponding decay channels and the cavity field, with bright interlayer excitons IX_B_ exhibiting higher coupling efficiency than gray excitons IX_G_ and phonon-assisted decay channels of momentum-dark excitons. This finding can be understood in the framework of dipolar selection rules in AB stacking: the wavevector of circularly polarized IX_B_ emission is collinear with the cavity which optimally enhances the respective decay channel. The enhancement is weaker for the decay channel of gray excitons IX_G_ with *z*-polarized in-plane emission. Momentum-dark excitons IX_L_, finally, exhibit the same Purcell enhancement as IX_G_ as they decay via the gray exciton channel though phonon-assisted spin-valley flipping processes.

All three decay channels responded consistently to cavity length detuning, as shown in Fig. 4c. At a cavity length of 35 μm, several cavity modes were resonant with the HBL emission peak thus enhancing all possible emission channels simultaneously. For cavity lengths smaller than 9 μm, however, the free spectral range of the cavity exceeded the linewidth of the HBL emission peak, rendering cavity-coupling sensitive to the spectral resonance condition. Open circles in Fig. 4c show the results for off-resonant configurations in accord with cavity-inhibited radiative decay. In contrast, the on-resonance data (measured with a dense spacing of data points for ~6–8 μm cavity lengths in Fig. 4c) reflect the effect of cavity-enhancement with anti-correlated trends for short and long decay components at smallest cavity lengths consistent with spectrally distinct channels. At a nominal separation of ~5 μm (gray circles), physical contact between the fiber and the extended mirror was presumably reached, preventing further reduction of the cavity mode volume.

The data recorded in contact of the fiber and the macro-mirror as well as all off-resonance data were discarded from the following analysis of the cavity-induced Purcell enhancement in the presence of pure dephasing^38^. On resonance, the generalized Purcell factor is *F*_P_ = (4*g*^2^/*γ*_fs_)/(*κ* + *γ*_fs_ + *γ*_d_), where *g* is the coupling rate of the emitter to the cavity, *κ* is the cavity decay rate, and *γ*_d_ is the dephasing rate of the emitter. Both *g* and *κ* vary as a function of the cavity length^29,39,40^. By taking the inhomogeneous linewidth *γ* = 55 meV deduced from the data in Fig. 2b as an upper bound to the dephasing rate in our system (i. e. using *γ*_d_ ≤ *γ*), we fitted each data set of Fig. 4c according to the model for the generalized Purcell enhancement (see Supplementary Note 4 for details). The resulting best fits, shown as solid lines in Fig. 4c, were obtained with free-space lifetimes of 5.8 ± 0.4, 39 ± 2, and 760 ± 40 ns for the three sets of data in the respective panels of Fig. 4c. These asymptotic values at infinite cavity length extracted from the model fit agree well with the decay times determined in confocal PL spectroscopy (data in Fig. 2b).

With this strong confidence in the correspondence between the free-space lifetime values extracted from the model of generalized Purcell enhancement and the decay times obtained in the absence of the cavity with confocal PL spectroscopy, the model allows now to extrapolate maximum Purcell enhancement $F_{\mathrm{P}}^{{\mathrm{max}}}$ that can be achieved at the peak wavelength of the HBL emission *λ* for a mirror separation of *λ*/2. The model yields $F_{\mathrm{P}}^{{\mathrm{max}}}$ of 2.9 ± 0.2 for the short and 1.7 ± 0.1 for both the intermediate and long lifetime channels, respectively. For the same limit of the intermirror spacing of *λ*/2 and a cavity volume of ~*λ*^3^, the model also quantifies the light-matter coupling strength *g* as 195 ± 9, 58 ± 3, and 13 ± 0.9 μeV for IX_B_, IX_G_, and phonon-assisted decay of momentum-dark excitons, respectively. These values, in good quantitative agreement with the absorption contrast in Fig. 1, are quite robust against variations in the dephasing rate, with *g* changing by <25% for *γ*_d_ in the range of 10–70 meV. At the same time light-matter coupling was sensitive to material and environmental characteristics with up to 50% changes in *g* and about 30% variations in the free-space PL lifetimes on different positions of the same flake and different flakes.

## Discussion

The values for the light-matter coupling strength *g* of interlayer excitons in our CVD-grown MoSe_2_-WSe_2_ HBL sample are two to three orders of magnitude smaller than the coupling rates reported for MLs TMDs^6–9^. This striking difference in light-matter coupling, fully consistent with the spatially indirect nature of interlayer excitons in HBL systems, yields tight constraints on the observation of interlayer exciton-polariton phenomena in the strong-coupling regime of HBL – cavity hybrids. To ensure *g* > *κ* + *γ*_d_ for strong-coupling, cavities with higher quality factors are readily available^41^, yet much improved HBL crystals and environmental conditions will be required to reduce dephasing. However, in view of radiatively limited linewidths achieved for ML TMDs by encapsulation with hexagonal boron nitride^42–45^, further progress towards the realization of dipolar exciton-polariton gases in cavity – van der Waals heterostructure systems seems feasible.

## Methods

### Chemical vapor deposition of vertical TMD heterobilayers

First, MoSe_2_ ML was grown by selenization of molybdenum trioxide (MoO_3_) powder. SiO_2_/Si substrate along with MoO_3_ powder boat were placed at the center of a chemical vapor deposition (CVD) furnace, which was heated to 750 °C in 15 min and held for 20 min. SiO_2_/Si substrate was facing down in close proximity with MoO_3_ powder. Selenium (Se) powder vaporized at 200 °C was used as Se source, and a mixture of argon and hydrogen (15% hydrogen) at 50 SCCM was used as the carrier gas. The as-grown MoSe_2_/SiO_2_/Si was then transferred to a separate CVD setup for subsequent WSe_2_ growth similar to the method of MoSe_2_. Specifically, selenization of tungsten oxide (WO_3_) was performed at 900 °C in the presence of 100 SCCM carrier gas. WSe_2_ would grow on top of MoSe_2_ from its edges, creating MoSe_2_/WSe_2_ vertical heterostructures. No additional treatment was necessary prior to WSe_2_ growth due to thermal removal of possible physisorbed molecule gases on MoSe_2_ during the transfer in air. As-grown heterostructures were studied in spectroscopy or transferred onto a mirror using polymer-supported wet transfer method. To this end polymethylmethacrylate (PMMA) was spin-coated on the heterostructure and lifted off in 1 M potassium hydroxide (KOH) in water. Finally, the PMMA-supported film with MoSe_2_-WSe_2_ vertical heterostructures on the mirror was rinsed in three cycles of water to remove possible KOH residue.

### Photoluminescence spectroscopy

PL experiments were performed in a lab-built cryogenic setup. The sample was mounted on piezo-stepping units (attocube systems ANPxy101 and ANPz102) for positioning with respect to a low-temperature objective (attocube systems LT-APO/NIR/0.81) or the cavity mode. The microscope was placed in a dewar with an inert helium atmosphere at a pressure of 20 mbar and immersed in liquid helium at 4.2 K. Excitation around 635–705 nm was performed with a wavelength-tunable supercontinuum laser (NKT SuperK Extreme and SuperK Varia) with repetition rates down to 2 MHz. In continuous-wave measurements, the PL was spectrally dispersed by a monochromator (Princeton Instruments Acton SP 2500) and recorded with a nitrogen-cooled silicon CCD (Princeton Instruments PyLoN). Time-resolved PL was detected with avalanche photodiodes (Excelitas SPCM-AQRH or PicoQuant *τ*SPAD).

### Scanning cavity microscopy

The cryogenic cavity was composed of a fiber micro-mirror and a macroscopic mirror with MoSe_2_-WSe_2_ vertical HBL on top. The macro-mirror was coated with ~30 nm of silver and a spacer layer of SiO_2_ with thickness designed to place the HBL at a field antinode. The effective radius of curvature of the central depression in the laser-machined fiber end facet was 136 μm. The facet was coated with ~50 nm silver and a protection layer of SiO_2_. Three translational degrees of freedom of the sample on the mirror were accessible by cryogenic positioners (attocube systems ANPxy101 and ANPz102) to provide both lateral scans and coarse-tuning of the cavity length. Cavity fine-tuning was achieved by displacing the fiber-mirror with an additional piezo. Excitation by a supercontinuum laser (NKT SuperK Extreme and SuperK Varia) at 635 nm was provided via the optical fiber and both transmission and PL were detected through the planar macro-mirror with the heterostructure on top. Two-dimensional scans were performed with a cavity length of ~22 μm resulting in a mode-waist of 3.2 μm for the excitation laser and a mode-waist of 3.7 μm for the detected PL ~880 nm.

## Supplementary information

Supplementary Information

## Data Availability

The data that support the findings of this study are available from the corresponding author on reasonable request.
