# Supplementary material for: Cavity-control of interlayer excitons in van der Waals heterostructures
Source: Nat Commun. 2019 Aug 16;10:3697. doi: 10.1038/s41467-019-11620-z (PMC6697722; doi:10.1038/s41467-019-11620-z)
Supplement: Supplementary file 1 — Supplementary Information [file 41467_2019_11620_MOESM1_ESM.pdf]

SUPPLEMENTARY INFORMATION:

Cavity-control of interlayer excitons in van der Waals heterostructures

Förg et al.

(Dated: July 25, 2019)

Supplementary Note 1: Theory of interlayer excitons in vertical transition metal dichalcogenide heterobilayers

A. Symmetry of transition metal dichalcogenide monolayer

The real space lattice of a transition metal dichalcogenide (TMD) monolayer (ML) crystal is shown in Supplementary Figure 1. Yellow and blue circles represent the positions of chalcogen and metal atoms, respectively.

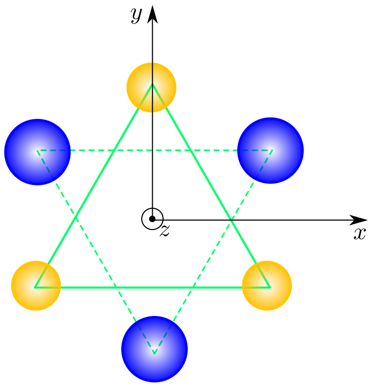

Supplementary Figure 1: Positions of atoms in the lattice of a transition metal dichalcogenide monolayer. Blue and yellow circles represent the positions of metal and chalcogen atoms, respectively.

To introduce the transformation rules for the wavefunctions, we follow Ref. [1] and (using $\cos 2\pi/3 = -1/2$) define the action of C_3 rotation as:

$$\psi(\boldsymbol{r}) \to D_{C_3}\psi(\boldsymbol{r}) = \psi(\boldsymbol{r}') = \psi(\hat{C}_3^{-1}\boldsymbol{r}), \quad \hat{C}_3^{-1} = \begin{pmatrix} -1/2 & \sqrt{3}/2 & 0 \\ -\sqrt{3}/2 & -1/2 & 0 \\ 0 & 0 & 1 \end{pmatrix}, \tag{1}$$

or

$$x' = -\frac{x}{2} - \frac{\sqrt{3}y}{2}, \quad y' = -\frac{y}{2} + \frac{\sqrt{3}x}{2}.$$

Here the operator $\hat{C}_3$ is the coordinate transformation, D_{C_3} is the function transformation. As a result, we obtain

$$D_{C_3}(x + \mathrm{i}y) = e^{\mathrm{i}\frac{2\pi}{3}}(x + \mathrm{i}y), \quad D_{C_3}(x - \mathrm{i}y) = e^{-\mathrm{i}\frac{2\pi}{3}}(x - \mathrm{i}y), \tag{2}$$

$$D_{C_3}(x - \mathrm{i}y)^2 = e^{\mathrm{i}\frac{2\pi}{3}}(x - \mathrm{i}y)^2, \quad D_{C_3}(x + \mathrm{i}y)^2 = e^{-\mathrm{i}\frac{2\pi}{3}}(x + \mathrm{i}y)^2,$$

in full agreement with the character table for the C_{3h} point group [1].

The positions of metal atoms, $\boldsymbol{R}_M$, and of the chalcogen atoms, $\boldsymbol{R}_X$, in the unit cell (projected on the ML plane) are given by

$$\boldsymbol{R}_M = \frac{a_0}{2}(1, 1/\sqrt{3}), \quad \boldsymbol{R}_X = \frac{a_0}{2}(1, -1/\sqrt{3}), \tag{3}$$

where a_0 is the lattice constant (see Supplementary Figure 1). The wavevectors of the $\boldsymbol{K}_\pm$ valleys are given by

$$\boldsymbol{K}_\pm = \frac{2\pi}{a_0}(\pm 2/3, 0). \tag{4}$$

Thus, the transformation rules for the Bloch factors $\exp(\mathrm{i}\boldsymbol{K}_\pm \boldsymbol{R}_{M,X})$ are given by

$$D_{C_3} \exp(\mathrm{i}\boldsymbol{K}_\pm R_M) = e^{\pm \mathrm{i}\frac{2\pi}{3}} \exp(\mathrm{i}\boldsymbol{K}_\pm R_M), \quad D_{C_3} \exp(\mathrm{i}\boldsymbol{K}_\pm R_X) = e^{\mp \mathrm{i}\frac{2\pi}{3}} \exp(\mathrm{i}\boldsymbol{K}_\pm R_X). \tag{5}$$

The valence band Bloch functions in $\boldsymbol{K}_\pm$ valleys are invariant if the center of point group transformation coincides with the center of the hexagon as in Supplementary Figure 1 [2, 3]. Therefore, the atomic orbital of the metal atoms of the valence band in the $\boldsymbol{K}_+$ ($\boldsymbol{K}_-$) valley transforms as a function with an angular momentum component $+2$ (-2). The correspondence between the representations of C_{3h} point group for different origin of coordinates (relevant for one valley of TMD ML) is given in Supplementary Table 1. For simplicity we denote the representations as Γ_n (rather than K_n), where $n = 1 \ldots 12$, despite the fact that these states are relevant to the K-points of the Brillouin zone.

B. Symmetry of vertical transition metal dichalcogenide heterobilayer

The schematic illustration of atomic registries in vertical TMD heterobilayers is shown in Supplementary Figure 2. In H-type heterobilayer, the layers are rotated by $180°$ with respect to each other, while in the R-type heterobilayers the rotation is absent. In the following we present the symmetry analysis of electronic states in vertical TMD heterobilayers and address the optical selection rules without phonon-assisted processes. To illustrate the group-theory approach we focus first on AB stacking. Successively, other registries are analyzed.

The symmetry of the heterobilayer with AB stacking (the metal atom in one layer is on top of the metal atom in the second layer) is described by the C_{3v} point symmetry group. The three fold rotation axis C_3 goes through the metals and there are three "vertical" (i.e., containing the axis) reflection planes σ_v which contain metal and chalcogen atoms. Apart from the operations described above and the identity, there are no other symmetry operations in point group for AB stacking. The horizontal reflection plane vanishes since A and B layers are different, and thus mirror-reflection axes are also absent.

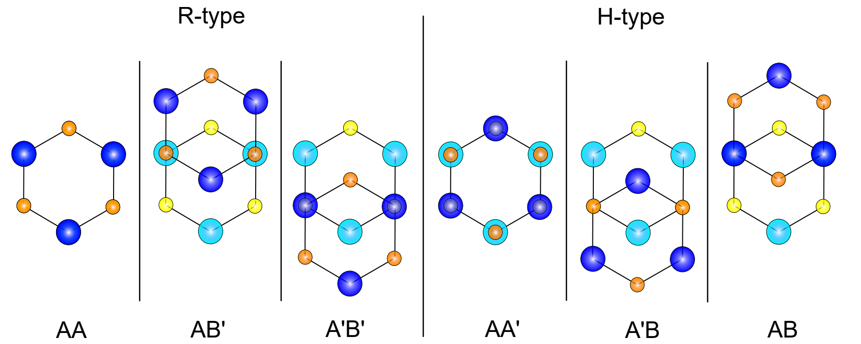

Supplementary Figure 2: High symmetry atomic registry for H- and R-type transition metal dichalcogenide heterobilayers. The cyan and yellow lattice represent the tungsten diselenide layer in which the holes reside. The large and small spheres represent the transition metal and the chalcogen atoms, respectively.

In the reciprocal space, due to π-rotation of the $MoSe_2$ layer with respect to the WSe_2 layer, the Brillouin zones are also rotated by π. The $\boldsymbol{K}_+$ valley of one monolayer is on top of the $\boldsymbol{K}_-$ valley of the other. In first approximation, the states are not hybridized due to weak coupling between the layers. The symmetry of the valley is C_3. Indeed, the three fold rotation remains, because $2\pi/3$ rotation transforms the $\boldsymbol{K}$-point to the equivalent one. On the other hand, all reflection planes vanish as the vertical reflection transforms $\boldsymbol{K} \to -\boldsymbol{K}$.

In order to establish the representations relevant for the bands, we determine the fixed point (origin) of the point group transformations. Whereas for a ML there is a freedom of choice (one can use one of three alternatives: (a) center of hexagon, (b) metal atom, (c) chalcogen atom), the origin for the bilayer is fixed by the stacking. For the AB stacking the origin is at the metal atom. Therefore, for the AB stacking the relevant column is (b) in Tab. 1. Thus, we have 2 $\boldsymbol{K}$-points ($\boldsymbol{K}_W - \boldsymbol{K}'_M$ and $\boldsymbol{K}'_W - \boldsymbol{K}_M$), each has two orbital conduction bands (CB) and two orbital valence bands (VB) stemming from the corresponding bands of $MoSe_2$ and WSe_2 MLs. In C_3 point group there are just three vector representations:

$$A\ (\Gamma_1):\ \text{conduction band orbital function (invariant)}, \tag{6}$$

$$E_1\ (\Gamma_2):\ \text{valence band orbital function in } \boldsymbol{K}_W - \boldsymbol{K}'_M\ (x+iy), \tag{7}$$

$$E_2\ (\Gamma_3):\ \text{valence band orbital function in } \boldsymbol{K}'_W - \boldsymbol{K}_M\ (x-iy), \tag{8}$$

the representations E_1 and E_2 are connected by the time-reversal. Moreover, there are just three spinor representations in C_3 point group:

$$\Gamma_4:\ (\uparrow), \quad \Gamma_5:\ (\downarrow), \quad \Gamma_6:\ (\text{either } +3/2 \text{ or } -3/2). \tag{9}$$

Supplementary Table 2 summarizes the representations for CB and VB states for the AB stacking.

By taking into account the multiplication rules

$$\Gamma_5 \times \Gamma_4^* = \Gamma_5 \times \Gamma_5 = \Gamma_3, \quad \Gamma_4 \times \Gamma_5^* = \Gamma_4 \times \Gamma_4 = \Gamma_2, \tag{10}$$

$$\Gamma_5 \times \Gamma_5^* = \Gamma_4 \times \Gamma_4^* = \Gamma_4 \times \Gamma_5 = \Gamma_1,$$

$$\Gamma_6 \times \Gamma_5^* = \Gamma_6 \times \Gamma_4 = \Gamma_3, \quad \Gamma_6 \times \Gamma_4^* = \Gamma_6 \times \Gamma_5 = \Gamma_2,$$

Supplementary Table 2 allows us to establish the selection rules for the interband transitions as:

- $\boldsymbol{K}'_W - \boldsymbol{K}_M$, topmost valence band ($WSe_2$) to bottom $\uparrow$ conduction band ($MoSe_2$) — z,
- $\boldsymbol{K}'_W - \boldsymbol{K}_M$, topmost valence band ($WSe_2$) to top $\downarrow$ conduction band ($MoSe_2$) — σ^-,
- $\boldsymbol{K}_W - \boldsymbol{K}'_M$, topmost valence band ($WSe_2$) to bottom $\downarrow$ conduction band ($MoSe_2$) — z,
- $\boldsymbol{K}_W - \boldsymbol{K}'_M$, topmost valence band ($WSe_2$) to top $\uparrow$ conduction band ($MoSe_2$) — σ^+.

Other stackings are analyzed in Tabs. 3 and 4.

For the AA' stacking the center of point group transformations is chosen in the center of hexagon and the selection rules are:

- $\boldsymbol{K}'_W - \boldsymbol{K}_M$, topmost valence band ($WSe_2$) to bottom $\uparrow$ conduction band ($MoSe_2$) — σ^-,
- $\boldsymbol{K}'_W - \boldsymbol{K}_M$, topmost valence band ($WSe_2$) to top $\downarrow$ conduction band ($MoSe_2$) — σ^+,
- $\boldsymbol{K}_W - \boldsymbol{K}'_M$, topmost valence band ($WSe_2$) to bottom $\downarrow$ conduction band ($MoSe_2$) — σ^+,
- $\boldsymbol{K}_W - \boldsymbol{K}'_M$, topmost valence band ($WSe_2$) to top $\uparrow$ conduction band ($MoSe_2$) — σ^-.

For the A'B stacking the center of point group transformations is chosen in the chalcogen atom and the selection rules are:

- $\boldsymbol{K}'_W - \boldsymbol{K}_M$, topmost valence band ($WSe_2$) to bottom $\uparrow$ conduction band ($MoSe_2$) — σ^+,
- $\boldsymbol{K}'_W - \boldsymbol{K}_M$, topmost valence band ($WSe_2$) to top $\downarrow$ conduction band ($MoSe_2$) — z,
- $\boldsymbol{K}_W - \boldsymbol{K}'_M$, topmost valence band ($WSe_2$) to bottom $\downarrow$ conduction band ($MoSe_2$) — σ^-,
- $\boldsymbol{K}_W - \boldsymbol{K}'_M$, topmost valence band ($WSe_2$) to top $\uparrow$ conduction band ($MoSe_2$) — z.

In the R-type stacking the point group symmetry is the same C_{3v}. For the AA case the rotation center should be chosen as the center of hexagon. Correspondingly,

- $\boldsymbol{K}_W - \boldsymbol{K}_M$, topmost valence band ($WSe_2$) to bottom $\uparrow$ conduction band ($MoSe_2$) — σ^+,
- $\boldsymbol{K}_W - \boldsymbol{K}_M$, topmost valence band ($WSe_2$) to top $\downarrow$ conduction band ($MoSe_2$) — z,
- $\boldsymbol{K}'_W - \boldsymbol{K}'_M$, topmost valence band ($WSe_2$) to bottom $\downarrow$ conduction band ($MoSe_2$) — σ^-,
- $\boldsymbol{K}'_W - \boldsymbol{K}'_M$, topmost valence band ($WSe_2$) to top $\uparrow$ conduction band ($MoSe_2$) — z.

For the AB' case the centers of hexagon in the W-based ML (hole layer) correspond to the metals Mo in the electron layer. Thus, the rotation center can be chosen as the metal atom in the W-based layer and the chalcogen atom in the Mo-based layer:

- $\boldsymbol{K}_W - \boldsymbol{K}_M$, topmost valence band ($WSe_2$) to bottom $\uparrow$ conduction band ($MoSe_2$) — z,
- $\boldsymbol{K}_W - \boldsymbol{K}_M$, topmost valence band ($WSe_2$) to top $\downarrow$ conduction band ($MoSe_2$) — σ^-,
- $\boldsymbol{K}'_W - \boldsymbol{K}'_M$, topmost valence band ($WSe_2$) to bottom $\downarrow$ conduction band ($MoSe_2$) — z,
- $\boldsymbol{K}'_W - \boldsymbol{K}'_M$, topmost valence band ($WSe_2$) to top $\uparrow$ conduction band ($MoSe_2$) — σ^+.

For the A'B' case the centers of hexagon in the W-based ML (hole layer) correspond to the dichalcogenides Se in the electron layer. Thus, the rotation center can be chosen as the chalcogen atom in the W-based layer and the metal atom in the Mo-based layer:

- $\boldsymbol{K}_W - \boldsymbol{K}_M$, topmost valence band ($WSe_2$) to bottom $\uparrow$ conduction band ($MoSe_2$) — σ^-,
- $\boldsymbol{K}_W - \boldsymbol{K}_M$, topmost valence band ($WSe_2$) to top $\downarrow$ conduction band ($MoSe_2$) — σ^+,
- $\boldsymbol{K}'_W - \boldsymbol{K}'_M$, topmost valence band ($WSe_2$) to bottom $\downarrow$ conduction band ($MoSe_2$) — σ^+,
- $\boldsymbol{K}'_W - \boldsymbol{K}'_M$, topmost valence band ($WSe_2$) to top $\uparrow$ conduction band ($MoSe_2$) — σ^-.

According to the D_{3d} point group which describes A'B $MoSe_2$-WSe_2 heterobilayers symmetries, only three spinor representations exist for the energy bands: Γ_6 for the top most WSe_2 VB, Γ_6 for the top $MoSe_2$ CB and Γ_5 (Γ_4) for the bottom $MoSe_2$ CB in the $K_M(K'_M)$ valley. These band symmetries determine the selection rules of the momentum-allowed transitions shown in Supplementary Figure 3. All other band symmetries are summarized in Supplementary Figure 3 and Supplementary Figure 4 for H-type and R-type stacking respectively.

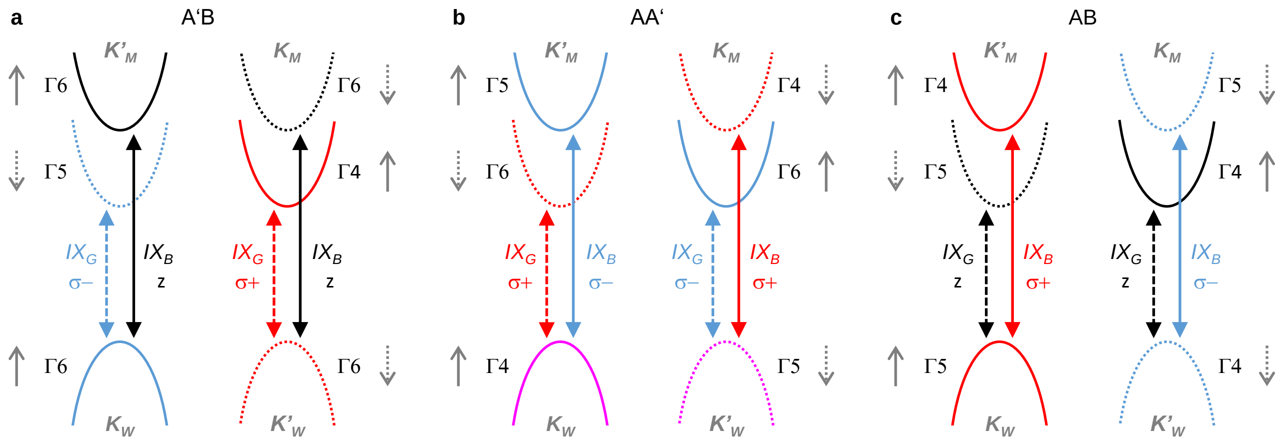

Supplementary Figure 3: Band symmetries and optical selection rules for the case of H-type stacking. **a**, $MoSe_2$ conduction band and WSe_2 valence band at K and K' forming the single-particle band diagram of a commensurate $MoSe_2$-WSe_2 heterobilayer in A’B stacking including band symmetries (Γ_4, Γ_5 and Γ_6 are the spinor representations) and dipolar transitions with $\sigma+$ (red), $\sigma-$ (blue) and z (black) polarization. **b** and **c**, Same for AA’ and AB stacking, respectively. Solid (dotted) bands represent spin up (down) electron polarization; solid (dashed) arrows represent spin-allowed (spin-forbidden) optical transitions. The energy bands are colored according to the polarization of the respective optical transitions except for the bands in magenta supporting both $\sigma+$ and $\sigma-$ transitions.

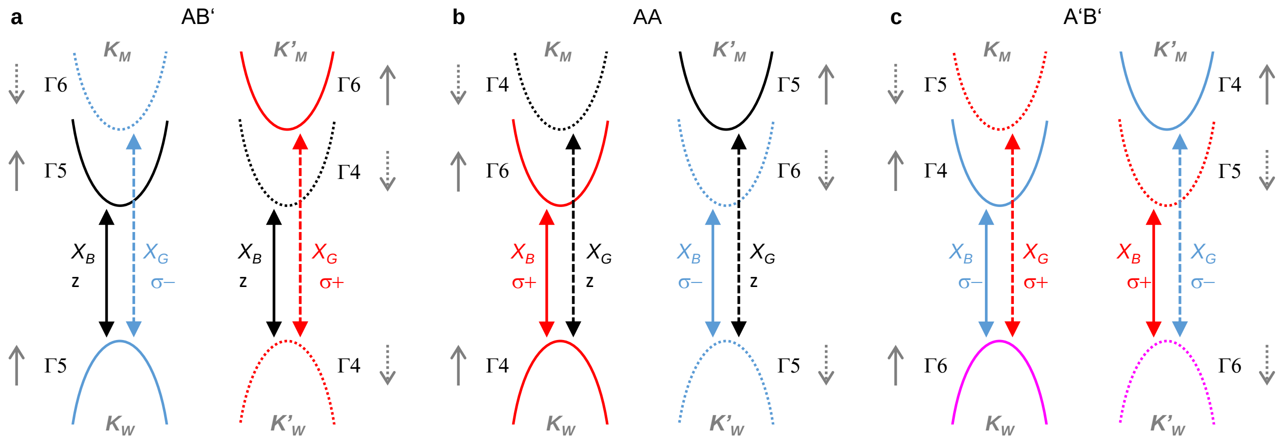

Supplementary Figure 4: Band symmetries and optical selection rules for the case of R-type stacking. The three panels display the relevant properties for AB’ (**a**), AA (**b**) and A’B’ (**c**) stacking order. The pictorial representation of different spins and polarizations is similar to Supplementary Figure 3

Supplementary Table 1: Correspondence between the representations of the C_{3h} point groups relevant for $\boldsymbol{K}_{\pm}$ in ML TMD. Both vector and spinor representations are given, $\uparrow$ and $\downarrow$ denote the spin-up $|1/2, +1/2\rangle$ and spin-down $|1/2, -1/2\rangle$ states. Only the topmost valence band ($\uparrow$ in $\boldsymbol{K}_+$ valley and $\downarrow$ in $\boldsymbol{K}_-$ valley) are relevant.

valley	band	(a) hexagon	(b) metal	(c) chalcogen
$\boldsymbol{K}_+$	valence	Γ_1	Γ_3	Γ_2
	conduction	Γ_2	Γ_1	Γ_3
$\boldsymbol{K}_-$	valence	Γ_1	Γ_2	Γ_3
	conduction	Γ_3	Γ_1	Γ_2
$\boldsymbol{K}_+$	valence $\uparrow$	Γ_7	Γ_{10}	Γ_{11}
	conduction $\uparrow$	Γ_{11}	Γ_7	Γ_{10}
	conduction $\downarrow$	Γ_9	Γ_8	Γ_{12}
$\boldsymbol{K}_-$	valence $\downarrow$	Γ_8	Γ_9	Γ_{12}
	conduction $\downarrow$	Γ_{12}	Γ_8	Γ_9
	conduction $\uparrow$	Γ_{10}	Γ_7	Γ_{11}

Supplementary Table 2: Symmetries of bands in $\boldsymbol{K}_W - \boldsymbol{K}'_M$ and $\boldsymbol{K}'_W - \boldsymbol{K}_M$ points of heterobilayer with AB stacking.

valley	band	representation
$\boldsymbol{K}'_W - \boldsymbol{K}_M$	valence	Γ_2
	conduction	Γ_1
$\boldsymbol{K}_W - \boldsymbol{K}'_M$	valence	Γ_3
	conduction	Γ_1
$\boldsymbol{K}'_W - \boldsymbol{K}_M$	valence $\downarrow$	Γ_4
	conduction $\uparrow$	Γ_4
	conduction $\downarrow$	Γ_5
$\boldsymbol{K}_W - \boldsymbol{K}'_M$	valence $\uparrow$	Γ_5
	conduction $\downarrow$	Γ_5
	conduction $\uparrow$	Γ_4

Supplementary Table 3: Symmetries of bands in $\boldsymbol{K}_W - \boldsymbol{K}'_M$ and $\boldsymbol{K}'_W - \boldsymbol{K}_M$ points of heterobilayer with AA' stacking (centers of hexagons coincide in the monolayers).

valley	band	representation
$\boldsymbol{K}'_W - \boldsymbol{K}_M$	valence	Γ_1
	conduction	Γ_2
$\boldsymbol{K}_W - \boldsymbol{K}'_M$	valence	Γ_1
	conduction	Γ_3
$\boldsymbol{K}'_W - \boldsymbol{K}_M$	valence $\downarrow$	Γ_5
	conduction $\uparrow$	Γ_6
	conduction $\downarrow$	Γ_4
$\boldsymbol{K}_W - \boldsymbol{K}'_M$	valence $\uparrow$	Γ_4
	conduction $\downarrow$	Γ_6
	conduction $\uparrow$	Γ_5

Supplementary Table 4: Symmetries of bands in $\boldsymbol{K}_W - \boldsymbol{K}'_M$ and $\boldsymbol{K}'_W - \boldsymbol{K}_M$ points of heterobilayer with A'B stacking (chalcogenes coincide).

valley	band	representation
$\boldsymbol{K}'_W - \boldsymbol{K}_M$	valence	Γ_2
	conduction	Γ_2
$\boldsymbol{K}_W - \boldsymbol{K}'_M$	valence	Γ_3
	conduction	Γ_3
$\boldsymbol{K}'_W - \boldsymbol{K}_M$	valence $\downarrow$	Γ_6
	conduction $\uparrow$	Γ_4
	conduction $\downarrow$	Γ_6
$\boldsymbol{K}_W - \boldsymbol{K}'_M$	valence $\uparrow$	Γ_6
	conduction $\downarrow$	Γ_5
	conduction $\uparrow$	Γ_6

Supplementary Table 5: Symmetries of bands in $\boldsymbol{K}_W - \boldsymbol{K}_M$ and $\boldsymbol{K}'_W - \boldsymbol{K}'_M$ points of heterobilayer with AA stacking (centers of hexagons coincide).

valley	band	representation
$\boldsymbol{K}_W - \boldsymbol{K}_M$	valence	Γ_1
	conduction	Γ_2
$\boldsymbol{K}_W - \boldsymbol{K}_M$	valence	Γ_1
	conduction	Γ_3
$\boldsymbol{K}_W - \boldsymbol{K}_M$	valence $\uparrow$	Γ_4
	conduction $\uparrow$	Γ_6
	conduction $\downarrow$	Γ_4
$\boldsymbol{K}'_W - \boldsymbol{K}'_M$	valence $\downarrow$	Γ_5
	conduction $\downarrow$	Γ_6
	conduction $\uparrow$	Γ_5

Supplementary Table 6: Symmetries of bands in $\boldsymbol{K}_W - \boldsymbol{K}_M$ and $\boldsymbol{K}'_W - \boldsymbol{K}'_M$ points of heterobilayer with AB' stacking.

valley	band	representation
$\boldsymbol{K}_W - \boldsymbol{K}_M$	valence	Γ_3
	conduction	Γ_3
$\boldsymbol{K}_W - \boldsymbol{K}_M$	valence	Γ_2
	conduction	Γ_2
$\boldsymbol{K}_W - \boldsymbol{K}_M$	valence $\uparrow$	Γ_5
	conduction $\uparrow$	Γ_5
	conduction $\downarrow$	Γ_6
$\boldsymbol{K}'_W - \boldsymbol{K}'_M$	valence $\downarrow$	Γ_4
	conduction $\downarrow$	Γ_4
	conduction $\uparrow$	Γ_6

Supplementary Table 7: Symmetries of bands in $\boldsymbol{K}_W - \boldsymbol{K}_M$ and $\boldsymbol{K}'_W - \boldsymbol{K}'_M$ points of heterobilayer with A'B' stacking.

valley	band	representation
$\boldsymbol{K}_W - \boldsymbol{K}_M$	valence	Γ_2
	conduction	Γ_1
$\boldsymbol{K}_W - \boldsymbol{K}_M$	valence	Γ_3
	conduction	Γ_1
$\boldsymbol{K}_W - \boldsymbol{K}_M$	valence $\uparrow$	Γ_6
	conduction $\uparrow$	Γ_4
	conduction $\downarrow$	Γ_5
$\boldsymbol{K}'_W - \boldsymbol{K}'_M$	valence $\downarrow$	Γ_6
	conduction $\downarrow$	Γ_5
	conduction $\uparrow$	Γ_4

Supplementary Note 2: Analysis of photoluminescence decay

A. Deconvolution procedure

Time-correlated photoluminescence (TCPL) decay was recorded with two different avalanche photodiodes (APDs) with 900 ps (Excelitas SPCM-AQRH) and 440 ps (PicoQuant τSPAD) response times in confocal spectroscopy and cavity experiments, respectively. The total heterobilayer (HBL) photoluminescence (PL) decay was recorded either without spectral filtering or within different spectral bands indicated by the bars in Supplementary Figure 5.

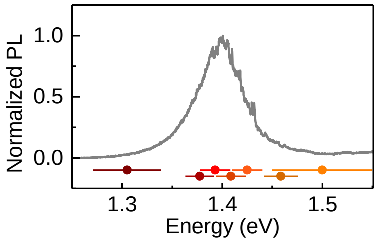

Supplementary Figure 5: Normalized HBL PL peak shown together with spectral windows used in time-correlated PL decay measurements.

The PL decay traces were modeled as a convolution of the APD instrument response function (IRF) and multi-exponential decay functions as follows:

$$I(t) = I_0 + A_0 \cdot e^{-2\cdot[(t-t_0)/w]^2} + \sum_{k=1}^{N} A_k \cdot e^{(-t/\tau_k)}, \tag{11}$$

where the first term I_0 quantifies the APD dark counts, the second term is the APD IRF approximated by a Gaussian with the temporal resolution w, and the third is the sum of N individual exponential decay channels k with amplitude A_k and characteristic decay time τ_k. The dark counts

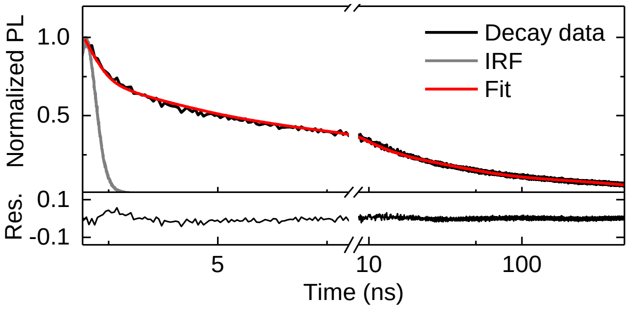

Supplementary Figure 6: Upper panel: Normalized time-correlated PL decay at a cavity length of 30 μm (black), experimental APD instrument response function (gray), and model fit with three exponential decay channels (red solid line with $\tau_1 = 4.3$ ns, $\tau_2 = 33$ ns, $\tau_3 = 660$ ns). Lower panel: Residuum of the fit.

and the response times w of both APDs were calibrated experimentally as in Supplementary Figure 6. The time t_0, set to the maximum of each TCPL trace, was an input parameter to the fits with the amplitudes A_k and the decay times τ_k as free fit parameters. A representative model fit to a TCPL trace (recorded for a cavity length of 30 μm) obtained with there decay channels is shown in Supplementary Figure 6.

B. Minimum number of decay channels

To identify the minimum number of channels required to approximate the multi-exponential decay of the HBL emission, the TCPL traces were fitted with a varying number of channels using Supplementary Equation 11. The number of possible channels was increased from two up to five. For each number this analysis was applied to the whole set of TCPL measurements from 35 μm down to 5 μm cavity length. The quality of each fitting procedure was assessed by the χ^2-value. An average χ^2-value for all analyzed cavity lengths is shown in Supplementary Figure 7a, where it decreased down to the measurement noise level for an increasing number of possible channels.

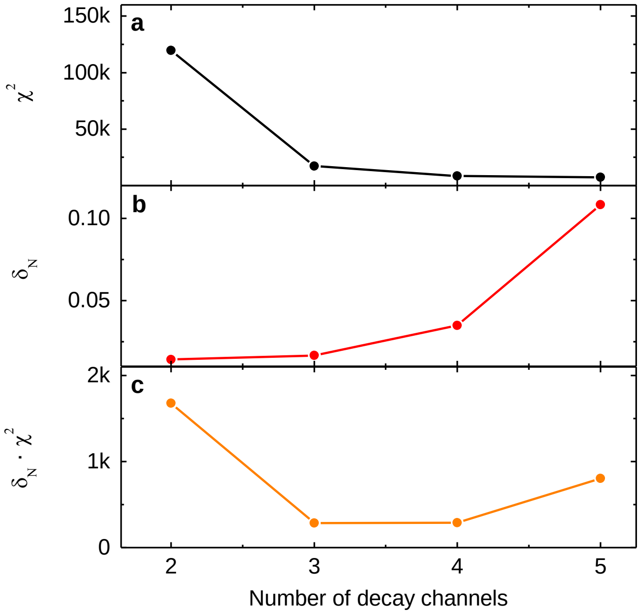

Supplementary Figure 7: **a**, Best-fit χ^2 average, obtained by averaging χ^2 values of multi-channel best-fits for all measurements at variable cavity lengths, as a function of the number of decay channels. **b**, Averaged and normalized parameter errors δ_N for the corresponding data. **c**, Product $\delta_N \cdot \chi^2$ of both error types. The minimum of the product at $N = 3$ indicates that three decay channels are best suited to approximate the multi-channel decay characteristics of the HBL peak in Fig. 1 of the main text.

The χ^2-value restricts the analysis to the quantity of the overlap of the best-fit function with the measured data, neglecting the possible errors of the individual free fit parameters. According to Supplementary Equation 11, each fitting procedure is characterized by a set of free fit parameters with respective errors. For best approximation of the TCPL data these errors should be minimized. Therefore, an averaged error was calculated for each fit using the errors of the individual free fit parameters. A mean value for all corresponding lifetime traces results in an overall error of the free fit parameters, δ_N. The corresponding errors are shown in Supplementary Figure 7b. The more possible decay channels contribute, the higher is the overall error δ_N of the free fit parameters. To establish a quantity that respects both types of errors, the product $\delta_N \cdot \chi^2$ was calculated. The result is shown in Supplementary Figure 7c. This product has its minimum for three decay channels, indicating that this description is best suited to approximate the dynamics of the HBL emission both in confocal and cavity-assisted TCPL spectroscopy.

Supplementary Note 3: Spectral decomposition of interlayer exciton photoluminescence

The HBL sample in our experiment corresponds to AB stacking in H-type registry (Supplementary Figure 3c). For this stacking, we obtain from the symmetry analysis above two photoactive zero-momentum interlayer excitons. Bright excitons, $\mathrm{IX_B}$, involve an unoccupied spin-up (spin-down) VB state in $\mathrm{WSe_2}$ at K (K') and an occupied spin-up (spin-down) CB state in $\mathrm{MoSe_2}$ at K' (K). Gray excitons, $\mathrm{IX_G}$, involve an unoccupied spin-up (spin-down) VB state in $\mathrm{WSe_2}$ at K (K') and an occupied spin-down (spin-up) CB state in $\mathrm{MoSe_2}$ at K' (K). These bright and gray exciton states are split by the CB spin-orbit splitting of $\mathrm{MoSe_2}$ and degenerate with their respective time-reversal counterparts. Additionally, finite-momentum interlayer excitons result from spin-like ($\mathrm{IX_L}$) and spin-unlike ($\mathrm{IX_U}$) combinations of unoccupied spin-up (spin-down) VB states in $\mathrm{WSe_2}$ at K (K') and occupied spin-up (spin-down) CB states in $\mathrm{MoSe_2}$ at K (K'). These momentum-dark states $\mathrm{IX_U}$ and $\mathrm{IX_L}$ with non-zero center-of-mass momentum are resonant with $\mathrm{IX_B}$ and $\mathrm{IX_G}$, respectively, and do not decay via direct radiative pathways. However, radiative recombination can occur via the bright and gray decay channels with assistance of phonons. The respective contribution of phonon-assisted decay to the PL appears as phonon-sidebands separated from the bare energy of momentum-dark excitons $\mathrm{IX_U}$ and $\mathrm{IX_L}$ by the energies of acoustic, optical, or combinations of multiple phonons [4, 5].

To model the total PL spectrum, we follow the procedure introduced for monolayers [4] and homobilayer [5]. On the blue side of the heterobilayer photoluminescence peak in the spectral range of $1.30 - 1.45$ eV, bright and gray excitons contribute with their respective zero photon line (ZPL) emission. In the absence of exchange interactions, the momentum-dark exciton reservoirs $\mathrm{IX_U}$ and $\mathrm{IX_L}$ are energetically degenerate with $\mathrm{IX_B}$ and $\mathrm{IX_G}$, respectively, which in turn are separated by the spin-orbit splitting of 25 meV in monolayer $MoSe_2$ [6, 7]. The higher-energy reservoir $\mathrm{IX_U}$, degenerate with $\mathrm{IX_B}$, is assumed to be void of population due to relaxation into the lower-energy momentum-dark reservoir $\mathrm{IX_L}$. Thus, the red side of the peak is composed of phonon sidebands of momentum-dark $\mathrm{IX_L}$ excitons that decay with the assistance of acoustic, optical and higher-order phonon processes [4, 5].

In order to simplify the analysis and reduce the number of relevant phonon modes, we employ the hole valley locking approximation [8–10] where the intervalley scattering of VB states is neglected and only CB scattering is considered. In this approximation, phonon-assisted radiative decay processes involve electron scattering with $MoSe_2$ phonons. The energies of optical and acoustic phonons with Γ-momentum and K-momentum in monolayer $MoSe_2$ were taken from Ref. [11]. In our sample, inhomogeneous broadening with a full-width at half-maximum (FWHM) of about 50 meV dominates the PL linewidth and therefore the zero-photon lines (ZPL) of $\mathrm{IX_B}$ and $\mathrm{IX_G}$ exciton transitions as well as the phonon sidebands of $\mathrm{IX_L}$ and $\mathrm{IX_U}$ states were modelled by Gaussians. Due to large inhomogeneous broadening, we neglect the expected deviations of ± 2 meV for $MoSe_2$ phonon energies in $MoSe_2$-WSe_2 heterobilayers. Moreover, since the energy differences between longitudinal optical (LO) and transverse optical (TO) as well as longitudinal acoustic (LA) and transverse acoustic (TA) phonons are negligible on the scale of the inhomogeneous broadening, we use in our decomposition analysis only one energy for acoustic phonons (18.3 meV) and one for optical phonons (32.8 meV) obtained as averages of LA and TA mode energies and LO, TO and and A_1 mode energies, respectively.

With these simplifications, we decompose the HBL peak in a best-fit procedure into individual contributions of $\mathrm{IX_B}$ and $\mathrm{IX_G}$ ZPLs and phonon sidebands (PS) of $\mathrm{IX_L}$ momentum-dark excitons. The results of two different fitting procedures are shown in the left and right panels of Supplementary Figure 8. In both approaches, we used a linear function to fit the background on the high energy side of the PL spectrum and allowed the fit procedure to determine the energy of $\mathrm{IX_B}$ as well as the amplitudes of Gaussians representing ZPLs of $\mathrm{IX_B}$ and $\mathrm{IX_G}$ and phonon sidebands of $\mathrm{IX_L}$ with a joint FWHM inhomogeneous linewidth. In the first approach (left panel of Sup-

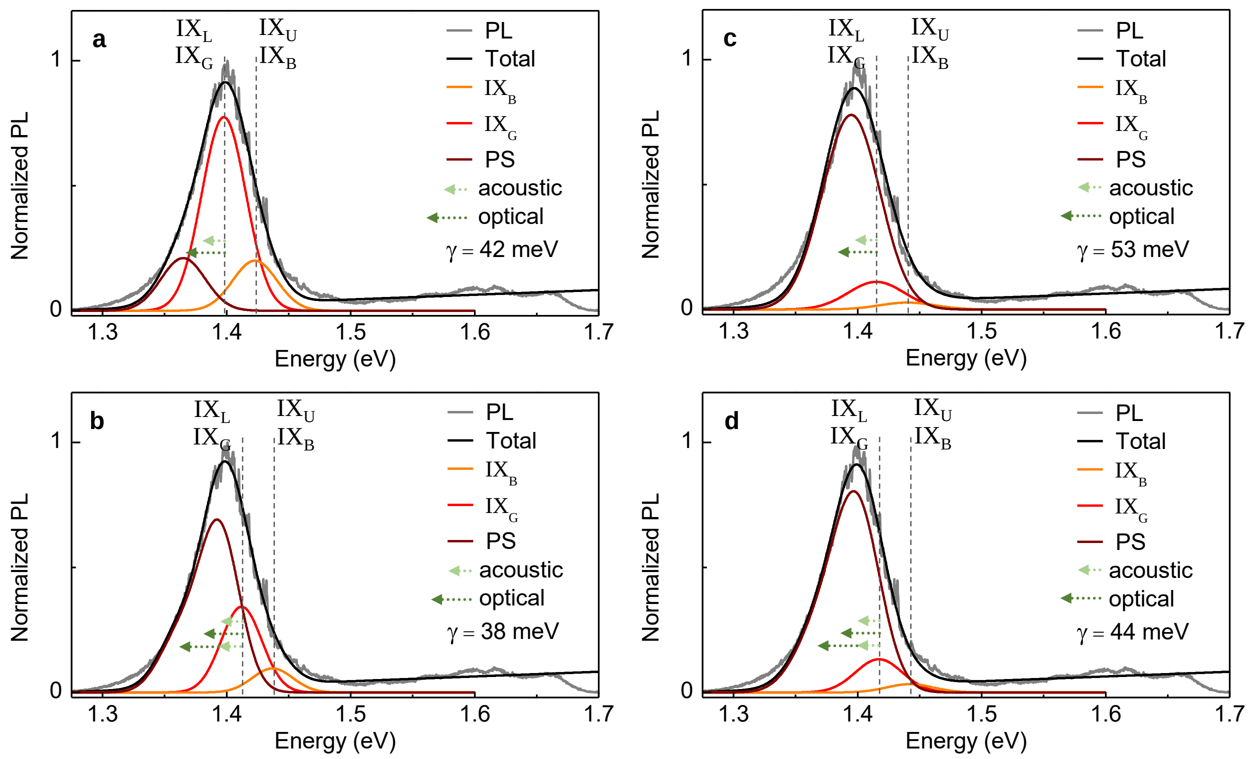

Supplementary Figure 8: Photoluminescence spectrum of $MoSe_2$-WSe_2 interlayer excitons (gray) with best fit (black solid line). Individual fit contributions zero-phonon lines of bright IX_B and gray IX_G excitons are shown as solid orange and red lines, respectively, and the sum of phonon sidebands of momentum-dark excitons IX_L are shown in brown. The reservoir of momentum-dark excitons IX_U is assumed to be void of population and thus does not contribute to the spectrum. First order acoustic and optical phonon processes are represented by light green and green arrows. **a**, Resulting fit with an un-constraint model and first order phonon processes. **b**, Same for first and second order phonon processes. **c**, Resulting fit with a constraint model and first order phonon processes. **d**, Same for first and second order phonon processes.

plementary Figure 8), we impose no constraints on the Gaussian amplitudes and perform best-fit decomposition with first order phonon processes only. The resulting best fit is shown in Supplementary Figure 8a. The fit yields a predominant contribution of IX_G (red solid line) to the total HBL peak which decreases when we take into account second order processes as in Supplementary Figure 8b. The contribution of IX_L phonon sidebands to the spectrum (brown solid line) in turn increases at the expense of IX_G ZPL in qualitative agreement with our TRPL data shown in Fig. 2b of the main text.

In the second approach, a quantitative agreement with TRPL data in Fig. 2b of the main text can be established by constraining the amplitudes of IX_B and IX_G, as shown in the right panel of Supplementary Figure 8. Using the fractions of the radiative energy from Fig. 2b, we decompose the PL spectrum with first order (Supplementary Figure 8c) and second order (Supplementary Figure 8d) phonon processes. Note that both approaches yield similar energies for IX_B and IX_G excitons at ~ 1.440 and 1.415 eV, respectively, and the FWHM linewidths are in good agreement with the inhomogeneous broadening of 55 meV determined from Fig. 2b of the main text.

Supplementary Note 4: Cavity-emitter coupling

Our cryogenic cavity was composed of a fiber micro-mirror and a macroscopic mirror with $MoSe_2$-WSe_2 vertical HBL on top. The macro-mirror was coated with ~ 30 nm of silver and a spacer layer of SiO_2 with thickness designed to place the HBL at a field antinode. The effective radius of curvature of the central depression in the laser-machined fiber end facet was 136 μm. The facet was coated with ~ 50 nm silver and a protection layer of SiO_2. Three translational degrees of freedom of the sample on the mirror were accessible by cryogenic positioners to provide both lateral scans and coarse-tuning of the cavity length. Cavity fine-tuning was achieved by displacing the fiber-mirror with an additional piezo. Excitation by a supercontinuum laser (NKT SuperK Extreme and SuperK Varia) at 635 nm was provided via the optical fiber and both transmission and PL were detected through the planar macro-mirror with the heterostructure on top. Two-dimensional scans were performed with a cavity length of ~ 22 μm resulting in a mode-waist of 3.2 μm for the excitation laser and a mode-waist of 3.7 μm for the detected PL around 880 nm. Transmission characteristics of the bare cavity as a function of cavity length shown in Supplementary Figure 9 were recorded with a supercontinuum laser.

To model the dependence of the cavity decay rate κ and the emitter-cavity coupling rate g on the cavity length L, we use the framework of a quantum well coupled to a two dimensional cavity [12], where:

$$\kappa = 2 \cdot \frac{1-\sqrt{R}}{\sqrt{R}} \frac{c}{n_c L_c}. \tag{12}$$

The cavity decay rate κ is extracted as the full-width at half-maximum linewidth of broadband transmission spectra recorded for an empty cavity (i.e. off $MoSe_2$-WSe_2 flakes) at a given cavity length. The values of κ we extract are consistent with a reflectivity coefficient of $R = 0.87$, which

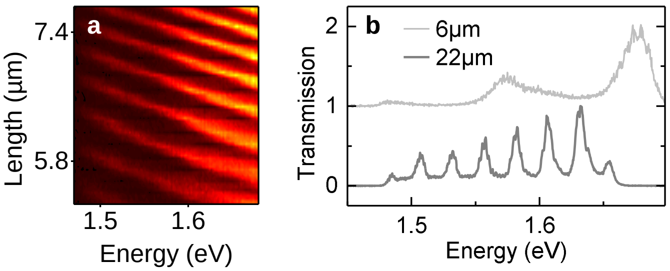

Supplementary Figure 9: Transmission characteristics of the cavity. **a**, Heatmap of the cavity transmission as a function of the cavity length. **b**, Transmission spectrum for a cavity length of 6 and 22 μm, offset for clarity.

allows to simplify Supplementary Equation 12 as:

$$\kappa(L) = \kappa_0 \cdot \frac{\lambda}{2L}, \tag{13}$$

where κ_0 is the cavity decay rate at a mirror separation of $\lambda/2$. For our cavity system we obtain $\kappa_0 = 410$ meV. Similar considerations for the collective coupling rate g_k yield for each individual channel:

$$g_k(L) = g_{0,k} \cdot \sqrt{\frac{\lambda}{2L}}. \tag{14}$$

In the next step we use $\gamma_{tot}/\gamma_{fs} = (\gamma_{fs} + \gamma_c)/\gamma_{fs} = 1 + F_p$, the ratio of the total decay rate in the cavity system γ_{tot} to the free-space decay rate γ_{fs}, together with the expression for the generalized Purcell factor $F_p = (4g^2/\gamma_{fs})/(\kappa + \gamma_{fs} + \gamma_d)$ [13, 14] to obtain the equation for the individual decay channels:

$$\gamma_{tot,k} = \gamma_{fs,k} \cdot \left(1 + \frac{4g_k^2/\gamma_{fs,k}}{\kappa + \gamma_{fs,k} + \gamma_d}\right),$$

with γ_d being the dephasing rate of the emitter. The functional dependence of the rate enhancement on the cavity length can be obtained by using Supplementary Equations 13 and 14. In a final step the rate enhancement is converted into a lifetime change via $\tau_{tot,k} = 1/\gamma_{tot,k}$ and $\widetilde{\tau}_k = 1/\gamma_{fs,k}$. The resulting fitting function takes $g_{0,k}$ and $\widetilde{\tau}_k$ as free fitting parameters.

Supplementary References

1. G. F. Koster, Properties of the thirty-two point groups, vol. 24 (The MIT Press, 1963).
2. A. Kormányos, V. Zólyomi, N. D. Drummond, P. Rakyta, G. Burkard, and V. I. Fal'ko, Monolayer MoS_2: trigonal warping, the γ valley, and spin-orbit coupling effects, Phys. Rev. B **88**, 045416 (2013).
3. A. Kormányos, G. Burkard, M. Gmitra, J. Fabian, V. Zólyomi, N. D. Drummond, and V. Fal'ko, k·p theory for two-dimensional transition metal dichalcogenide semiconductors, 2D Mater. **2**, 022001 (2015).
4. J. Lindlau, C. Robert, V. Funk, M. Förg, L. Colombier, A. Neumann, T. Taniguchi, K. Watanabe, M. M. Glazov, X. Marie, et al., Identifying optical signatures of momentum-dark excitons in monolayer transition metal dichalcogenides, arXiv:1710.00988 (2017).
5. J. Lindlau, M. Selig, A. Neumann, L. Colombier, J. Förste, V. Funk, M. Förg, J. Kim, G. Berghäuser, T. Taniguchi, et al., The role of momentum-dark excitons in the elementary optical response of bilayer WSe_2, Nat. Commun. **9**, 2586 (2018).
6. K. Kośmider, J. W. González, and J. Fernández-Rossier, Large spin splitting in the conduction band of transition metal dichalcogenide monolayers, Phys. Rev. B **88** (2013).
7. A. Ciarrocchi, D. Unuchek, A. Avsar, K. Watanabe, T. Taniguchi, and A. Kis, Polarization switching and electrical control of interlayer excitons in two-dimensional van der waals heterostructures, Nat. Photon. **13**, 131 (2019).
8. C. Mai, A. Barrette, Y. Yu, Y. G. Semenov, K. W. Kim, L. Cao, and K. Gundogdu, Many-body effects in valleytronics: Direct measurement of valley lifetimes in single-layer MoS_2, Nano Lett. **14**, 202 (2013).
9. C. Mai, Y. G. Semenov, A. Barrette, Y. Yu, Z. Jin, L. Cao, K. W. Kim, and K. Gundogdu, Exciton valley relaxation in a single layer of WS_2 measured by ultrafast spectroscopy, Phys. Rev. B **90**, 041414 (2014).
10. J. Kim, C. Jin, B. Chen, H. Cai, T. Zhao, P. Lee, S. Kahn, K. Watanabe, T. Taniguchi, S. Tongay, et al., Observation of ultralong valley lifetime in WSe_2/MoS_2 heterostructures, Sci. Adv. **3**, e1700518 (2017).
11. Z. Jin, X. Li, J. T. Mullen, and K. W. Kim, Intrinsic transport properties of electrons and holes in monolayer transition-metal dichalcogenides, Phys. Rev. B **90**, 045422 (2014).
12. V. Savona, L. C. Andreani, P. Schwendimann, and A. Quattropani, Quantum well excitons in semiconductor microcavities: Unified treatment of weak and strong coupling regimes, Solid State Commun. **93**, 733 (1995).

13. E. Hinds, Cavity quantum electrodynamics, in Advances in Atomic, Molecular, and Optical Physics (Elsevier, 1990), vol. 28, pp. 237–289.
14. A. Auffeves, D. Gerace, J.-M. Gérard, M. F. Santos, L. C. Andreani, and J.-P. Poizat, Controlling the dynamics of a coupled atom-cavity system by pure dephasing, Phys. Rev. B **81**, 245419 (2010).
